# Lycopene-rich diets modulate HDL functionality and associated inflammatory markers without affecting lipoprotein size and distribution in moderately overweight, disease-free, middle-aged adults: A randomized controlled trial

**DOI:** 10.3389/fnut.2022.954593

**Published:** 2022-08-01

**Authors:** Jane McEneny, Sarah-Louise Henry, Jayne Woodside, Susan Moir, Amelia Rudd, Nick Vaughan, Frank Thies

**Affiliations:** ^1^School of Medicine, Dentistry and Biomedical Sciences, Centre for Public Health, Queen University, Belfast, United Kingdom; ^2^School of Medicine, Medical Sciences and Nutrition, University of Aberdeen, Aberdeen, United Kingdom

**Keywords:** lycopene, high density lipoprotein, functionality, serum amyloid A, dietary intervention, tomato-rich diet

## Abstract

**Background:**

The consumption of lycopene-rich foods may lower cardiovascular disease (CVD) risk. Lycopene circulates in the blood bound to lipoproteins, including high-density lipoproteins (HDLs). Preliminary data from our group showed that increased consumption of tomato-based food or lycopene supplement in middle-aged subjects led to functional changes to HDL's sub-fractions, HDL_2_ and HDL_3_. These changes were also associated with a decrease in serum amyloid A (SAA), potentially enhancing their anti-atherogenic properties.

**Objective:**

We carried out a comprehensive randomized controlled intervention trial with healthy middle-aged volunteers to assess whether the consumption of tomato-based foods or lycopene supplements affects HDL functionality and associated inflammatory markers, and lipoprotein subfractions size and distribution.

**Design:**

Volunteers (225, aged 40–65 years) were randomly assigned to one of three dietary intervention groups and asked to consume a control diet (low in tomato-based foods, <10 mg lycopene/week), a lycopene-rich diet (224–350 mg lycopene/week), or the control diet with a lycopene supplement (70 mg lycopene/week). HDL_2_ and HDL_3_ were isolated by ultracentrifugation. Compliance was monitored by assessing lycopene concentration in serum. Systemic and HDL-associated inflammation was assessed by measuring SAA concentrations. HDL functionality was determined by monitoring paraoxonase-1 (PON-1), cholesteryl ester transfer protein (CETP), and lecithin cholesterol acyltransferase (LCAT) activities. The lipoprotein subfractions profile was assessed by NMR.

**Results:**

Lycopene in serum and HDL significantly increased following consumption of both the high tomato diet and lycopene supplement (*p* ≤ 0.001 for both). Lycopene, either as a tomato-rich food or a supplement, enhanced both serum- and HDL_3_-PON-1 activities (*p* ≤ 0.001 and *p* = 0.036, respectively), while significantly reducing HDL_3_-SAA-related inflammation (*p* = 0.001). Lycopene supplement also significantly increased HDL_3_-LCAT activity (*p* = 0.05), and reduced the activity of both HDL_2_- and HDL_3_-CETP (*p* = 0.005 and *p* = 0.002, respectively). These changes were not associated with changes in the subclasses distribution for all lipoprotein fractions or the size of lipoprotein subclasses.

**Conclusion:**

Our results showed that dietary lycopene can significantly enhance HDL functionality, without associated changes in particle size and distribution, by modulating the activity of HDL-associated enzymes. Concomitantly, dietary lycopene significantly decreased serum- and HDL_3_-associated SAA, confirming that SAA may represent a sensitive inflammatory biomarker to dietary change.

**Clinical Trial Register:**

(https://www.isrctn.com), ISRCTN34203810.

## Introduction

Results from observational studies (1–4) have shown inverse associations between the consumption of tomato or lycopene and the risk of cardiovascular disease (CVD) and related mortality. However, intervention studies with tomato-based products or lycopene supplements focusing on conventional and non-conventional markers of CVD risk have generated inconsistent results. Some trials have shown that lycopene can reduce pro-inflammatory mediators and oxidative stress (5), and modulate some cardiovascular markers (6), namely, blood pressure (7), and lipid profile (8). Other studies, however, found no significant effects on blood lipids (9, 10) and blood pressure (11). A recent systematic review reported no significant differences between lycopene intervention and control groups for blood pressure and lipids (total cholesterol, low-density lipoprotein (LDL) cholesterol, high-density lipoprotein (HDL) cholesterol, and triglycerides), even in subgroup analyses for individuals with elevated risk factor concentrations at baseline (12). Results from a comprehensive intervention trial carried out in our group showed no effect of a 12-week intervention with a tomato-rich diet (35–50 mg lycopene/day) or lycopene supplement (10 mg/day) on conventional CVD risk markers (13). However, preliminary analysis in a subgroup of the participant from that study showed that increased lycopene intake using supplements or by dietary means over 12 weeks reduced serum amyloid A (SAA) content in serum and HDL. These changes were associated with a concomitant improvement in HDL functionality, as measured by the activity of HDL-associated enzymes such as paraoxonase 1 (PON-1), lecithin cholesterol acyl transferase (LCAT), and cholesterol ester transfer protein (CETP), potentially enhancing HDL anti-atherogenic properties (14), suggesting novel mechanisms by which lycopene could exert cardiovascular protection. Indeed, numerous studies have identified an inverse relationship between HDL concentration and CVD risk (15, 16). HDL, in addition to its central role in the reverse transport of cholesterol, has important antioxidant and anti-inflammatory properties. The lowering of SAA we observed in our subgroup analysis is particularly interesting, as SAA in HDL renders these lipoprotein particles dysfunctional (17, 18), with a reduction of PON-1 and LCAT activities (19, 20). However, these results needed to be confirmed in the full sample size. Furthermore, whether these changes are associated with changes in lipoprotein classes distribution and size remained to be determined.

## Methods

### Study population

The study was approved by the North of Scotland Research Ethics Committee (07/S0801/32) and conducted in concordance with CONSORT Guidelines (21) and details of the study design and CONSORT flow diagram have been previously published (13). We conducted a 16-week intervention trial involving 225 healthy men and women (40–65 years, 18.5 < BMI <35 kg/m^2^, sedentary or moderately active) randomized into three treatment groups: a control diet group (equivalent to 70 mg lycopene per week), a lycopene-rich diet group (high in tomato-based food and equivalent to 224–350 mg lycopene per week), and a lycopene supplement group (equivalent to 70 mg lycopene per week). Each of the diets was practical and realistic for individuals to achieve, with minor alterations to their usual lifestyle. Participants were provided with tomato-based products widely available from the main UK food retailers. Aside from the tomato servings supplied to the participant, the participants were instructed not to alter their food intake, apart from the prescribed changes, and to maintain their usual level of physical activity and lifestyle. Tomato-based food intake was restricted in the lycopene-supplemented group and the control group. All volunteers were not allowed to consume any of the forbidden foods listed below, but were able to consume up to 1 portion of tomato soup, tomato juice, or tomato sauce per week, and either (a) up to 4 raw tomatoes/24 cherry tomatoes per week or (b) up to 1 portion of tomato ketchup per week. Forbidden foods included pasta; canned tomatoes; cooked tomatoes; tomato paste; tomato puree; pizza; salsa; chutney; canned beans/spaghetti/ravioli, etc., in tomato sauce; barbecue sauce; brown sauce; pink grapefruit; guava; watermelon or apricots. Following a 4-week run-in period in which all participants consumed the control diet, volunteers were assigned to one of the above three groups for 12 weeks. Compliance was determined by analysis of serum lycopene concentration and by assessing the consumption of tomato-based foods through the use of a food diary at baseline, during the intervention, and at the end of the intervention. Individuals with signs of the metabolic syndrome, e.g., if they possessed three or more of the following conditions: fasting plasma glucose >6.1 mmol/L, triglyceride >1.7 mmol/L, low HDL-cholesterol (<1.04 mmol/L for men, <1.29 mmol/L for women), hypertension (>130/85 mmHg), and central obesity (waist circumference >102 cm for men, >88 cm for women), or if they had moderate hypercholesterolemia. Individuals were excluded if they suffered from CVD, diabetes, or asthma, or if they had thyroid or eating disorders, and systolic and diastolic blood pressures >160 and >99 mmHg, respectively. Subjects taking any medication known to affect any dependent variable being measured, those with a high habitual intake of tomato-based food (>5 servings per week) or those regularly consuming nutritional supplements were also excluded. All measurements were performed four times, before the run-in period, at baseline, during, and after the intervention. The samples obtained from the volunteers were anonymized and coded, and the people who assessed the outcomes (research assistants) were blinded after assignment to interventions. During each visit, fasting blood samples were collected, and weight, waist circumference, and blood pressure were measured. Compliance was assessed by measuring serum lycopene concentrations and by analyzing a weekly checklist of tomato-based foods consumed.

### Isolation of serum HDL subfractions

HDL_2_ and HDL_3_ were isolated from serum by rapid ultracentrifugation as previously described (22), using a three-step procedure; a crude (c) HDL was isolated by a 2-h rapid sedimentation method, and then subfractionated into HDL_2_ and HDL_3_ by two, 2-h sequential rapid flotation ultracentrifugation procedures. The concentrations of HDL_2_ and HDL_3_-associated total protein, apolipoprotein AI (apoAI), and SAA were reported using a dilution factor due to the process whereby the subfractions were isolated from serum. Initially, 1.2 ml serum/plasma was used to isolate the HDL subfractions, and collectively, 1.6 ml of sample was retrieved (0.8 ml HDL_2_ plus 0.8 ml HDL_3_). Taking into consideration the initial volume and volumes retrieved (1.2 ml/1.6 ml), the dilution factor applied to non-standardized (absolute) HDL_2_ and HDL_3_ concentrations was 1.33.

### Lycopene concentration

Lycopene concentration was measured in serum and HDL_2_ and HDL_3_ by reverse-phase high-performance liquid chromatography as previously described (23).

### ApoAI, SAA, IL-6, and hsCRP determination

Apolipoprotein AI (apoAI) concentration in the HDL subfractions (HDL_2_ and HDL_3_) was determined using single radial immunodiffusion as previously described (24). SAA, in serum and associated with HDL_2_ and HDL_3_, was analyzed by an ELISA (Invitrogen UK, KHA0011), as per the manufacturer's instructions. Analysis was performed using a Grifols TRITURUS system (Italy). Interleukin-6 (IL-6) was determined by ELISA (R&D Systems). High-sensitivity C-reactive protein (hsCRP) was measured at the Department of Clinical Biochemistry at Aberdeen Royal Infirmary using a standardized automated procedure (Behring nephelometry system).

### Protein concentration

Protein concentration within HDL_2_ and HDL_3_ was determined spectrophotometrically using the Biorad assay (BioRad 500-006, Biorad Hemel Hempstead, UK) as per the manufacturer's instructions. Total protein concentration was utilized to standardize SAA, PON-1, CETP, and LCAT within HDL_2_ and HDL_3_.

### PON-1, LCAT, and CETP activities

Paraoxonase-1 (PON-1) activity was measured by monitoring the hydrolysis of phenylacetate using a modification of the method of Hasselwander et al. (25) as previously described (14). The activity of CETP and LCAT was measured in HDL_2_ and HDL_3_ using commercially available fluorometric assays, as per the manufacturer's instructions (CETP catalog no. RB-CETP; LCAT catalog no. RB-LCAT; Roar Biomedical, NY, USA).

### Determination of lipoprotein classes concentration and size distribution by NMR

During each visit, 12-h fasted blood samples were taken from the antecubital fossa vein. Serum was prepared after centrifuging blood samples at 800 g at 4°C for 15 min and stored at −80°C until analysis. All samples were analyzed in a single batch to reduce variability. Very low-density lipoprotein (VLDL), LDL, and HDL subclasses concentrations and size in serum were determined by NMR (Liposcience Inc., Raleigh, USA). Data on intermediate-density lipoprotein (22.7–27 nm) are not reported in this article. The diameter range of the lipoprotein subclasses is shown in Table 1.

**Table 1:** Diameter range of lipoprotein subclasses.

Lipoprotein	Diameter (nM)
VLDL/Chylomicrons	
VLDL Particles (total)	
Large VLDL	>60
Medium VLDL	35–60
Small VLDL	27–35
LDL	
LDL Particles (total)	
Large LDL	21.2–23.0
Small LDL (total)	18.0–21.2
Medium small LDL	19.8–21.2
Very small LDL	18.0–19.8
HDL	
HDL particles (total)	
Large HDL	8.8–13.0
Medium HDL	8.2–8.8
Small HDL	7.3–8.2

## Results

### Subject characteristics

Baseline characteristics of the participants were comparable with regard to age, body mass index, blood pressure, and arterial stiffness (Table 1). More women than men were recruited; however, the proportion of women was similar in all the three groups. The slight imbalance in the number of subjects allocated per group was previously discussed (13).

### Lycopene concentration in serum and HDL subfractions

Serum lycopene concentration was similar across the groups at baseline, but significantly increased after 12-week intervention in both high tomato and lycopene supplement groups, compared to the low tomato group (13). Lycopene concentration in HDL_2_ and HDL_3_ subfractions was similar across the groups (*p* > 0.05, Table 2). Within-group analysis showed that lycopene content in HDL_2_ significantly increased following the high tomato diet (*p* < 0.001) compared to baseline. However, these changes were not significantly different compared from those observed in the other groups (*p* = 0.294). Both lycopene interventions significantly increased lycopene concentration in HDL_3_ by 54.7 and 23.4% for the tomato-rich diet and lycopene supplement, respectively, compared to the low tomato group (*p* = 0.005).

**Table 2:** Participant characteristics at baseline, by the intervention group^a^.

	High Tomato *n = 81*	Lycopene *n =* 68	Control *n =* 76	*P* ^b^
Age (years)	51.0 (0.7)^c^	51.1 (0.9)	51.1 (0.7)	0.889
Sex (n)				
Men Women	35 46	28 40	30 46	0.878^e^
Current smokers (n)	5	6	1	
BMI (kg/m^2^)	26.4 (0.5)	26.7 (0.5)	26.8 (0.5)	0.831
Waist circumference (cm)	88.1 (1.2)	89.0 (1.4)	88.2 (1.3)	0.907
Systolic BP (mm Hg)	129.4 (1.8)	130.8 (2.1)	130.8 (1.7)	0.953
Diastolic BP (mm Hg)	78.8 (1.4)	79.6 (1.3)	79.0 (1.1)	0.380
Pulse wave velocity (m/s)	8.20 (0.25)	8.34 (0.29)	8.32 (0.19)	0.605
Stiffness index (m/s)^d^	8.2 (0.3)	7.7 (0.3)	7.6 (0.2)	0.272
Alcohol (g/day)	12.0 (1.2)	11.1 (1.2)	13.0 (1.4)	0.479

a BP, blood pressure; BMI, body mass index.

b Differences between groups were assessed by using two-factor ANOVA (p < 0.05).

c Mean (SEM) (all such values).

d Calculated by using pulse contour analysis.

e Pearson's chi-squared test.

### Protein, apoA1, CRP, and SAA concentrations in serum and HDL fractions

Total protein and apoA1 in HDL_2_ and HDL_3_ were similar at baseline and remained unchanged after intervention in all the groups (not shown). None of the interventions affected hsCRP serum concentrations (13). However, serum hsCRP and SAA concentrations were significantly positively associated at baseline (Spearman's rho 0.513, *p* < 0.001). A similar association was observed between serum hsCRP and HDL_3_-SAA concentrations (Spearman's rho = 0.438, *p* < 0.001). These positive associations were also retained after each intervention. The effect of the interventions on SA concentrations in serum and HDL subfractions is shown in Table 3. Serum-SAA concentration was similar across the groups at baseline, but significantly decreased by 12% following a 12-week intervention with lycopene supplement compared with the other groups (*p* = 0.046). None of the interventions significantly affected SAA levels in HDL_2_. However, HDL_3_-SAA concentration significantly decreased (*p* < 0.001) in the lycopene supplement (−25.7%) and high tomato (−8.3%) groups, compared with the low tomato group, for which HDL_3_-SAA concentration significantly increased after intervention from baseline.

**Table 3:** Lycopene concentration in HDL_2_, HDL_3_, and serum following 12-week intervention with either a low tomato diet, lycopene supplement, or high tomato diet.

		*N*	Baseline	Wk 12	Change at wk12	Pre vs. post *P*-value
***Serum***	Low tomato diet	76	344 (22.4)	338 (26.4)^a^	−5.0 (-0.82, 0.071)^a^	0.765
	Lycopene supplement	68	395 (32.0)	863 (48.2)^b^	469 (389, 549)^b^	<0.001
	High tomato diet	81	427 (23.7)	1,146 (53.8)^c^	719 (646, 793)^c^	<0.001
	Between group *P*-value		0.068	<0.001	<0.001	
***HDL**_**2**_*	Low tomato diet	76	1.11 (0.11)	1.16 (0.18)	0.05 (-0.288, 0.391)	0.261
	Lycopene supplement	68	1.06 (0.12)	1.42 (0,19)	0.36 (-0.03, 0.721)	0.170
	High tomato diet	81	0.92 (0.11)	1.29 (0.17)	0.37 (0.041, 0.700)	<0.001
	Between group *P*-value		0.633	0.475	0.294	
***HDL**_**3**_*	Low tomato diet	76	0.14 (0.01)	0.14 (0.01)^a^	0.01 (-0.01, 0.04)^a^	0.824
	Lycopene supplement	68	0.14 (0.01)	0.18 (0.01)^a^	0.03 (0.01, 0.06)^a^	0.010
	High tomato diet	81	0.15 (0.01)	0.23 (0.02)^b^	0.08 (0.04, 0.12)^b^	<0.001
	Between group *P*-value		0.170	<0.001	0.006	

Results are mean (SEM), apart from change from baseline which are presented as mean (95% CI). Lycopene concentration is expressed as nmol/L in serum and as nmol/mg protein in HDL fractions. Differences in concentration changes at week 12. From baseline between groups were assessed by using two-factor ANOVA (p ≤ 0.05). Paired samples t-test was used to assess pre vs. post, with significance level set p ≤ 0.05. Values with different superscript letters are statistically significantly different (LSD post-hoc test).

### PON, LCAT, and CETP in HDL fractions

The effects of the interventions on PON-1, LCAT, and CETP activities in HDL fractions are summarized in Tables 4–6, respectively. At baseline, PON-1 and LCAT activities were similar for both HDL_2_ and HDL_3_ fractions while CETP activity was significantly lower in HDL_2_ from the high tomato group compared with the low tomato group (230 ± 5.29 vs. 256 ± 8.25, *p* = 0.025). None of the lycopene interventions significantly affected PON-1 and LCAT activities in HDL_2_ (*p* = 0.484 and *p* = 0.382 for change from baseline, respectively, Tables 4, 7). However, PON-1 activity significantly increased by 7.8 and 7.5% in HDL_3_ after a high tomato diet and lycopene supplement interventions, respectively (*p* = 0.036). Similar results were found for PON-1 activity in serum (*p* < 0.001, Table 7). Both interventions with lycopene diets tended to induce an increase in LCAT activity in HDL_3_ compared to baseline (significantly for the lycopene supplement, *p* = 0.05). After the 12-week intervention, CETP activity in HDL_2_ tended to be lower in both lycopene supplement groups compared to the low tomato group, significantly for the lycopene supplement group (*p* = 0.005, Table 6). HDL_3_-CETP activity significantly decreased after the 12-week intervention compared to baseline in both lycopene-rich groups (*p* < 0.001 for both lycopene supplement and high tomato diet). Between-group analysis of differences in concentration changes at week 12 from baseline showed that both lycopene interventions significantly lowered HDL_3_-CETP activity compared to the low tomato group (*p* = 0.002).

**Table 4:** PON-1 activity in serum, HDL_2_, and HDL_3_ following 12-week intervention with either a low tomato diet, lycopene supplement, or high tomato diet.

		*N*	Baseline (U/L)	WK 12 (U/L)	Change at WK 12 (U/L)	Pre Vs. post *P*-Value
SERUM	Low tomato diet	76	57,942 (2,108)	57,954 (2,065)^a^	12.4 (741)^a^	0.987
	Lycopene supplement	68	52,864 (2,204)	66,147 (3,558)^b^	13,497 (3,686)^b^	0.001
	High tomato diet	81	55,552 (2,297)	59,174 (1,742)^b^	3,622 (1,420)^c^	0.013
	Between group *P*-value		0.284	0.053	<0.001	
HDL_2_	Low tomato diet	69	1.88 (0.97)	2.09 (1.12)	0.21 (0.02, 0.41)	0.117
	Lycopene supplement	59	2.02 (1.80)	2.35 (1.87)	0.33 (−0.27, 0.93)	0.074
	High tomato diet	63	2.84 (2.51)	2.59 (2.63)	−0.25 (−0.69, 0.18)	0.058
	Between group *P*-value		0.832	0.418	0.484	
HDL_3_	Low tomato diet	69	2.30 (1.54)	2.25 (1.49)	−0.06 (−0.18, 0.06)^a^	0.240
	Lycopene supplement	59	2.42 (1.51)	2.50 (1.40)	0.11 (−0.04, 0.25)^b^	0.162
	High tomato diet	63	2.45 (1.44)	2.52 (1.41)	0.13 (−0.04, 0.29)^a, b^	0.005
	Between group *P*-value		0.806	0.182	0.036	

Results are expressed as mean (SEM) apart from change at week 12 which are presented as mean (95% CI). PON-1 activity in serum and in HDL_2_ and HDL_3_ is expressed as U/L and U/mg protein, respectively. Differences in concentrations at baseline, week 12 at week concentration changes at week 12 from baseline between groups were assessed by using two-factor ANOVA. Paired samples t-test was used to assess pre vs post (p ≤ 0.05). Values with different superscript letters are statistically significantly different (LSD post-hoc test).

**Table 5:** LCAT activity in HDL_2_ and HDL_3_ following 12-week intervention with either a low tomato diet, lycopene supplement, or high tomato diet.

		*N*	Baseline (470/390)	WK 12 (470/390)	Change at WK 12 (470/390)	Pre vs. post *P*-value
HDL_2_	Low tomato diet	69	5.24 (0.26)	5.28 (0.27)	0.04 (−0.06, 0.14)	0.919
	Lycopene supplement	59	5.79 (0.28)	5.85 (0.26)	0.06 (−0.05, 0.16)	0.161
	High tomato diet	63	5.15 (0.26)	5.09 (0.26)	−0.06 (−0.16, 0.04)	0.770
	Between group *P*-value		0.277	0.184	0.382	
HDL_3_	Low tomato diet	69	0.37 (0.02)	0.37 (0.02)	−0.00 (−0.01, 0.01)^a^	0.333
	Lycopene supplement	59	0.37 (0.02)	0.39 (0.02)	0.01 (0.00, 0.02)^b^	0.006
	High tomato diet	63	0.37 (0.02)	0.38 (0.02)	0.04 (−0.00, 0.01)^a, b^	0.123
	Between group *P*-value		0.952	0.577	0.050	

Results are expressed as mean (SEM) apart from change at week 12 which are presented as mean (95% CI). LCAT activity in HDL_2_ and HDL_3_ is expressed as 470:390 ratio and 470:390 ratio/mg protein, respectively. Differences in concentration changes at week 12 from baseline between groups were assessed by using one-factor ANOVA, with post-hoc LSD. Paired samples t-test was used to assess pre vs. post, with significance level set p ≤ 0.05. Values with different superscript letters are statistically significantly different (p < 0.05).

**Table 6:** CETP activity in HDL_2_ and HDL_3_ following 12-week intervention with either a low tomato diet, lycopene supplement, or high tomato diet.

		*N*	Baseline	WK 12	Change at WK 12	Pre vs. post *P*-value
HDL_2_	Low tomato diet	69	1.07 (0.45)	1.08 (0.44)	0.01 (−0.01, 0.03)^a^	0.382
	Lycopene supplement	59	1.11 (0.43)	1.04 (0.40)	−0.07 (−0.13, −0.01)^b^	0.004
	High tomato diet	63	0.94 (0.45)	0.93 (0.44)	−0.01 (−0.04, 0.01)^a, b^	0.095
	Between group *P*-value		0.026	0.008	0.005	
HDL_3_	Low tomato diet	69	2.55 (2.04)	2.93 (2.68)	0.39 (0.09, 0.69)^a^	0.714
	Lycopene supplement	59	2.61 (2.90)	1.84 (2.36)	−0.77 (−1.35, 0.20)^b^	<0.001
	High tomato diet	63	2.09 (1.82)	1.84 (2.01)	−0.25 (−0.57, 0.08)^b^	<0.001
	Between group *P*-value		0.143	0.036	0.002	

Results are expressed as mean (SEM) apart from change at week 12 which are presented as mean (95% CI). CETP activity in HDL_2_ and HDL_3_ is expressed as μmol/L and μmol/mg protein, respectively. Differences in concentration changes at week 12 from baseline between groups were assessed by using one-factor ANOVA, with post hoc LSD. Paired samples t-test was used to assess pre vs. post, with significance level set p ≤ .0.05. Values with different superscript letters are statistically significantly different (p < 0.05).

**Table 7:** SAA concentration in serum, HDL_2_, and HDL_3_ following 12-week intervention with either a low tomato diet, lycopene supplement, or high tomato diet.

		*N*	Baseline	Wk 12	Change at Wk 12	Pre vs. post *P*-value
*Serum*	Low tomato diet	76	18,005 (15,307)	20,461 (19,082)	2456 (−494, 5407)^a^	0.192
	Lycopene supplement	68	17,095 (16,465)	14,277 (15,355)	−2,819 (−5,326, −310)^b^	0.029
	High tomato diet	81	15,258 (13,644)	15,337 (14,205)	79 (−2,514, 2,671)^a, b^	0.656
	Between group *P*-value		0.605	0.097	0.046	
*HDL_2_*	Low tomato diet	69	2.84 (2.51)	2.59 (2.63)	−0.25 (−0.69, 0.18)	0.113
	Lycopene supplement	59	3.02 (2.77)	2.90 (3.97)	−0.12 (−0.97, 0.74)	0.725
	High tomato diet	63	2.84 (2.51)	2.59 (2.63)	−0.25 (−0.69, 0.18)	0.195
	Between group *P*-value		0.676	0.300	0.221	
*HDL_3_*	Low tomato diet	69	2.55 (2.04)	2.93 (2.68)^a^	0.39 (0.09, 0.69)^a^	0.001
	Lycopene supplement	59	2.61 (2.90)	1.84 (2.36)^b^	−0.77 (−1.35, 0.20)^b^	0.009
	High tomato diet	63	2.09 (1.82)	1.84 (2.01)^b^	−0.25 (−0.57, 0.08)^c^	0.092
	Between group *P*-Value		0.372	0.039	<0.001	

Results are expressed as mean (SEM) apart from change at week 12 which are presented as mean (95% CI). SAA concentration is expressed as μg /L in serum and as μg/mg protein in HDL fractions. Differences in concentration changes at week 12 from baseline between groups were assessed by using two-factor ANOVA. Paired samples t-test was used to assess pre vs post (p ≤ 0.05). Values with different superscript letters are statistically significantly different (LSD post-hoc test).

### Effects of interventions on lipoprotein subclasses distribution and sizes

The HDL subclasses distribution was similar between the groups at baseline and was not affected by the intervention (Table 8). VLDL and LDL profiles also remained unchanged after intervention for all the study groups (not shown). None of the dietary interventions significantly affected the size of lipoprotein classes (Table 9).

**Table 8:** HDL subclass distribution in response to 12-week intervention with high tomato, lycopene, and control diets.

		High tomato *n* = 81	Lycopene *n* = 68	Low tomato *n* = 76	*P^b^*	*P^c^*
HDL particles (Total)	Baseline^a^	32.7 (31.5, 33.8)^d^	31.6 (30.4, 32.7)	31.1 (30.2, 32.1)	0.117	
(μmol/l)	Wk12	32.5 (31.4, 33.5)	31.7 (31.5, 33.0)	31.8 (30.8, 32.9)		0.258
Large HDL particles	Baseline	8.8 (7.8, 9.7)	8.3 (7.4, 9.3)	8.5 (7.7, 9.4)	0.819	
(μmol/l)	Wk12	8.6 (7.7, 9.5)	8.3 (7.4, 9.3)	8.7 (7.9, 9.5)		0.701
Medium HDL particles	Baseline	3.7 (2.9, 4.5)	3.1 (2.3, 4.0)	3.1 (2.2, 4.0)	0.542	
(μmol/l)	Wk12	3.7 (2.9, 4.4)	3.7 (2.5, 4.8)	3.1 (2.4, 3.8)		0.535
Small HDL particles	Baseline	20.2 (18.9, 21.6)	20.1 (19.0, 21.3)	19.5 (18.3, 20.8)	0.706	
(μmol/l)	Wk12	20.1 (18.8, 21.5)	19.8 (18.4, 21.0)	20.1 (18.9, 21.2)		0.398

a Differences at baseline were assessed by using one-factor ANOVA on log-transformed values.

b Differences in concentration changes at week 6 from baseline between groups were assessed by using one-factor ANOVA.

c Differences in concentration changes at week 12 from baseline between groups were assessed by using one-factor ANOVA.

d Median; interquartile range in parentheses (all such values).

**Table 9:** Lipoprotein size in response to 12-week intervention with high tomato, lycopene, and control diets^a^.

		High tomato *n* = 81	Lycopene *n* = 68	Control *n* = 76	*P^b^*	*P^c^*
VLDL size (nm)	Baseline	49.20 (1.28)	46.99 (1.00)	48.42 (1.07)	0.935	
	Wk12^b^	48.97 (0.98)	50.00 (1.95)	49.30 (1.10)		0.865
LDL size (nm)	Baseline	21.50 (0.82)	21.32 (0.96)	21.40 (0.87)	0.356	
	Wk12	21.44 (0.81)	21.40 (0.97)	21.43 (0.95)		0.931
HDL size (nm)	Baseline	9.20 (0.06)	9.21 (0.07)	9.24 (0.06)	0.892	
	Wk12	9.21 (0.06)	9.20 (0.07)	9.26 (0.06)		0.785

a Data are the mean (SEM).

b Differences at baseline were assessed by using one-factor ANOVA on log-transformed values.

c Differences in size changes from baseline between the dietary intervention groups were assessed by using one-factor ANOVA.

## Discussion

This is the first comprehensive study identifying a specific dietary component, lycopene, able to significantly modulate activities related to HDL functionality. Our results confirm our preliminary results showing that dietary lycopene beneficially affects PON-1 activity in serum and HDL_3_ (14). These changes were associated with an increase in lycopene HDL content. PON-1 is an enzyme associated with HDL, particularly HDL_3_ (26), which, *via* its proton-donating properties, inactivates peroxidized lipids removed from apolipoprotein B-containing lipoproteins (27). Our results support previous findings in a diabetic rat model, which showed that lycopene restored PON-1 activity to normal levels (28). Human trials with diets rich in carotenoids also support our findings. In a small acute intervention, an increase in postprandial PON-1 activity concomitant to increased serum carotenoid concentrations was observed after ingestion of a Mediterranean-type meal in healthy subjects (29). Similarly, a diet rich in fruit and vegetables has been shown to increase PON-1 activity in type-2 diabetic subjects compared to a Western-style diet (30). Similar results were obtained after 1-year intervention with a traditional Mediterranean diet in people with a high risk of cardiovascular disease (31) and the authors suggested that bioactive compounds present in the diet, including carotenoids, could induce local antioxidant functions, preserve other dietary antioxidants in HDL lipoproteins, and protect PON1 against oxidative modifications or enhance its function.

Lecithin cholesterol acyltransferase (LCAT) is another enzyme closely associated with HDL_3_ (32). We observed an increase in LCAT-HDL_3_ after a 12-week intervention in both treatment groups, but only significant for the lycopene supplement group. Other interventions with a traditional Mediterranean diet or diet rich in fruit and vegetable has also shown improvement in LCAT activity (30, 33). An increase in LCAT activity would provide health benefits, considering that LCAT, and being involved in the maturation of HDL in the reverse cholesterol transport process (34), also functions as an antioxidant by accepting/donating protons *via* its active serine residue and is able to scavenge and neutralize free radicals that have been removed by HDL from LDL (24). LCAT is very sensitive to oxidative attacks (35) and lycopene may contribute to maintaining LCAT as non-oxidized and functional. Lycopene, through its ability to quench free radicals *via* proton donation (36), could donate electrons to LCAT, thus recycling LCAT's proton-donating site, overall increasing LCAT's anti-antioxidant activity. Daniels et al. (30) suggested that a carotenoid-enrichment in HDL could be responsible for the increase in activity of LCAT in HDL_3_ observed in subjects with type 2 diabetes following a high fruit and vegetable diet.

Cholesteryl ester transfer protein (CETP) plays a central role in cholesterol transfer from HDL to triglyceride-poor lipoproteins, and subsequently to the liver, and CETP activity is often increased in high-cardiovascular-risk states (ref). Therefore, the decrease in HDL_2_-CETP after lycopene supplementation (−5.5%) and the decrease in HDL_3_-CETP activity after intervention with a high tomato and a lycopene supplement (−5.9 and −6.9%, respectively) could be protective, as it may contribute to maintaining HDL homeostatic function. Unfortunately, we were unable to measure CETP activity in serum. However, it is likely that we would have observed a similar trend as CETP is majorly associated with HDL (37). Other interventions with carotenoid-rich diets showed reductions in HDL_3_-CETP activity in hypertensive subjects and in HDL_2_-CETP activity in elderly subjects (38). 1-year intervention with a traditional Mediterranean diet enriched in olive oil also showed a slight (−2.9%) but significant reduction in HDL CETP activity (31). The mechanisms by which lycopene could influence CETP activity are unclear, but could involve a reduction in CETP expression in the liver and adipose tissue. Unfortunately, due to sample availability, we were not able to determine the CETP content in serum and in HDL fractions.

Interestingly, the changes observed in enzymatic activities associated with HDL functionality were negatively associated with a 25.7 and 8.3% reduction in SAA concentration in HDL_3_ in the lycopene supplement and high tomato groups, respectively, while HDL_3_-SAA significantly increased following consumption of a low tomato diet. This suggests that increased lycopene intake beneficially lowers SAA-related inflammation. Like PON-1 and LCAT, SAA is particularly associated with HDL_3_ (39), therefore, any effects would be less readily identifiable in HDL_2_, which explains the lack of significant effect of the intervention observed in that particular HDL subfraction. SAA is an acute-phase protein that associates with HDL, rendering these particles dysfunctional, with reduced reverse transport of cholesterol (40), antioxidant (41), and anti-inflammatory (26) capabilities. Therefore, reducing SAA HDL content may be beneficial for HDL functionality. However, SAA renders HDL dysfunctional during a severe inflammatory response, when SAA can become the main apolipoprotein present on HDL (42) and the changes in SAA content might only be an indication of lycopene anti-inflammatory effects, and not related to HDL functionality. Lycopene has the ability to minimize the production and secretion of SAA from the liver and adipocytes (43, 44), which reduces systemic and HDL-related inflammation (44), which might explain the results observed.

The effects on HDL functionality and SAA observed were not associated with changes in HDL_2_ and HDL_3_ apoA1 content, which remained unchanged after intervention in all the groups (results not shown). This is in contradiction with previous findings reporting a significant increase in apoA1 concentration in subjects with type 2 diabetics who had consumed 200 g of raw tomatoes daily for 8 weeks (45). SAA concentration is inversely associated with apoA1 concentration (26, 46) while no change in apoA1 concentration following a reduction in SAA has also been reported (30). It is possible that apoA1 may have been redistributed within the HDL particles, thus meaning that there was no overall net change in apoA1 concentration. HDL can carry different numbers of apoA1 molecules (27), therefore, the loss of SAA from one specific HDL particle may allow apoA1 to be redistributed to another HDL particle, without changing the overall apoA1 HDL concentration.

The changes observed were also not associated with a concomitant change in lipoprotein subclass distribution and size. To our knowledge, this is the first time the effects of dietary lycopene on lipoprotein subclass distribution and size have been described. Carotenoids, including lycopene, circulate in the plasma bound to lipoproteins, and despite the significant enrichment in lycopene concentration both in serum and HDL, the particle subclasses and sizes remained unchanged. Results from subcohorts of the PREDIMED trial using NMR (47) or Lipoprint technology (31) showed an increase in large LDL/HDL and HDL fractions, respectively, after intervention with a Mediterranean diet in a population at high cardiovascular risk. However, our dietary interventions were minimal in comparison, and carried out on healthier subjects.

The main strengths of this study were excellent compliance, low dropout rates, and a relatively large sample size (*n* = 225) and appropriate comparators (low and high tomato diets and low tomato diets with lycopene supplement). Tomatoes are also rich in other phytochemicals and minerals which may potentially also alter HDL function and SAA content. However, the effects being more pronounced and predominant in the lycopene group compared with the high tomato diet strongly suggest that lycopene was responsible for improving HDL functionality and decreasing HDL-associated SAA content. The lycopene supplementation level and the dietary intervention were physiologically relevant and perfectly achievable. To our knowledge, no previous trial compared appropriately a lycopene supplement with a high-tomato diet, using an appropriate control arm. We measured the activities of three key enzymes (CETP, PON-1, and LCAT) and an inflammatory marker associated with HDL (SAA). However, we could not assess fully all aspects of HDL functionality, such as HDL cholesterol efflux capacity. We could also not determine the HDL mass content of the enzymes, and CETP and LCAT activities in serum. Further studies, namely, full HDL lipidomic and proteomic profile, are warranted to investigate the mechanisms by which dietary lycopene improves HDL function and whether these properties convey cardio protective effects.

## Data Availability Statement

The raw data supporting the conclusions of this article will be made available by the authors, without undue reservation.

## Ethics Statement

The studies involving human participants were reviewed and approved by North of Scotland Research Ethics Committee (07/S0801/32). The patients/participants provided their written informed consent to participate in this study.

## Author contributions

FT designed the randomized controlled trial and drafted the article. FT and JM designed lipoprotein and high-density lipoprotein experiments. JM and JW supervised S-LH. AR and NV conducted the trial. S-LH, AR, NV, and SM conducted laboratory analyses. S-LH, FT, JM, and JW analyzed data. All authors read and approved the final manuscript.

## Funding

This research was commissioned by the Food Standards Agency (grant number No. 2038) and funded by the Policy Research Programme in the Department of Health and the Food Standards Agency. FT received funding from the Scottish Government (RESAS).

## Conflict of interest

The authors declare that the research was conducted in the absence of any commercial or financial relationships that could be construed as a potential conflict of interest.

## Publisher's note

All claims expressed in this article are solely those of the authors and do not necessarily represent those of their affiliated organizations, or those of the publisher, the editors and the reviewers. Any product that may be evaluated in this article, or claim that may be made by its manufacturer, is not guaranteed or endorsed by the publisher.

## Author disclaimer

The views expressed are not necessarily those of the Department of Health.

## Abbreviations

CETP: cholesteryl ester transfer protein
CVD: cardiovascular disease
HDL: high-density lipoprotein
LCAT: lecithin cholesterol acyl transferase
LDL: low-density lipoprotein
PON-1: paraoxonase 1
SAA: serum amyloid A
VLDL: very low-density lipoprotein.
